# Effectiveness of Attachment-Based Interventions for Maltreated Children’s Well-Being: A Systematic Review

**DOI:** 10.1007/s10567-026-00556-8

**Published:** 2026-02-03

**Authors:** Kajung Hong, Rubi Alvarez-Rodriguez, Miguel T. Villodas

**Affiliations:** 1 San Diego State University/University of California San Diego Joint Doctoral Program in Clinical Psychology, San Diego, CA USA; 2 Department of Psychology, San Diego State University, San Diego, CA USA; 3 Child and Adolescent Services Research Center, San Diego, CA USA

**Keywords:** Systematic review, Attachment-based interventions, Parenting interventions, Child maltreatment

## Abstract

Many parenting interventions have been developed and implemented to mitigate the adverse effects of child maltreatment on children’s well-being. Intervention approaches rooted in attachment theory have the potential to improve the psychosocial and health outcomes of maltreated children by addressing parenting behaviors and caregiver–child relationships. However, few review studies have focused on assessing the impact of attachment-based parenting interventions on maltreated children’s psychosocial outcomes. The current systematic review synthesized findings from 30 attachment-based intervention studies (*N* = 1,423) that examined maltreated children’s emotional, behavioral, physiological, cognitive, and health outcomes post-interventions. The overall effectiveness varied across interventions, with many studies constrained by small sample sizes and reporting nonsignificant findings. Notably, short-term, attachment-based interventions using in-the-moment coaching tailored to families’ needs and administered by experienced providers tended to result in improved child outcomes. We conclude with suggestions for future directions in attachment-based intervention research.

## Introduction

Child maltreatment (CM) is a prevalent public health concern with enduring negative consequences for children’s well-being (Mehta et al., 2023; U.S. Department of Health and Human Services et al., 2025). Although parenting interventions based on cognitive behavioral and social learning theories are widely used to reduce the risk of CM and address its detrimental effects, parenting interventions based on attachment theory offer important perspectives on improving parenting behaviors and parent–child relationships to promote maltreated children’s psychosocial development. Indeed, substantial evidence supports the effectiveness of attachment-based interventions for improving parenting behaviors and caregiver-child attachment security (Kohlhoff et al., 2022; Mountain et al., 2017). However, few review studies have examined the effectiveness of attachment-based interventions on maltreated children’s emotional, behavioral, physiological, and cognitive outcomes, and the findings were mixed (Bakermans-Kranenburg et al., 2023; Barlow et al., 2015; Grube & Liming, 2018; Jugovac et al., 2022; Kerr & Cossar, 2014). The current systematic review synthesized literature on children’s emotional, behavioral, physiological, cognitive, and health outcomes of attachment-based parenting intervention.

## Effects of Child Maltreatment on Children’s Well-Being

CM affects one in four children (Massullo et al., 2023), leading to enduring developmental consequences. CM is one of the strongest predictors of several mental disorders and behavioral problems, such as depression, anxiety, criminality, and suicidality across the lifespan (e.g., Angelakis et al., 2019; Basto-Pereira & Farrington, 2022; Thomas et al., 2019). Individuals with CM histories exhibit earlier onset, higher severity, more comorbidities, and poorer psychopathology treatment outcomes across diagnostic categories (Lippard & Nemeroff, 2020). CM is also associated with adverse physical health consequences (Vizard et al., 2022), including dysregulated cortisol stress reactivity (Schär et al., 2022) and changes in DNA methylation (Cecil et al., 2020; Parade et al., 2021). Additionally, CM negatively impacts brain development (Teicher et al., 2016), posing a major risk for worse cognitive and educational outcomes in adolescence and adulthood (Strathearn et al., 2020; Su et al., 2019). As a result, the estimated lifetime costs associated with CM exceed $800,000 per victim (Peterson et al., 2018).

## Parenting Interventions Among Caregiver-Child Dyads Exposed to Child Maltreatment

The U.S. Centers for Disease Control and Prevention data indicate that approximately 90% of CM is perpetrated by parents or caregivers (Massullo et al., 2023; U.S. Department of Health and Human Services et al., 2022). With CM primarily occurring in maladaptive caregiver-child relationships, targeting caregivers is crucial to ensure the safety and well-being of their children and prevent CM and its deleterious consequences. As a result, over 20 parenting interventions using different approaches have been developed, tested, and adapted for children exposed to CM (e.g., Bergsund et al., 2023; Landers et al., 2018; Toth et al., 2013). One study performed a systematic review and meta-analysis, which aimed to find the effectiveness of relationship-based interventions for maltreated youth on improving caregiver–child behaviors (Bergsund et al., 2023). The study reviewed 81 publications with 20 different parenting interventions, including Parent–Child Interaction Therapy (PCIT) (Eyberg & Robinson, 1982), Attachment and Biobehavioral Catch-up (ABC) (Dozier et al., 2002, 2006), Video Feedback Intervention to Promote Positive Parenting (VIPP) (Juffer, 1993; Juffer et al., 2008), Filial Therapy (FT) (Guerney, 1964), and Child–Parent Psychotherapy (CPP) (Lieberman, 2004). The meta-analysis with a smaller subset of studies that included observational data found a large effect in improving observed parent interactive behavior, a moderate effect in improving child attachment, and a small effect in improving child interactive behavior.

In evidence-based parenting interventions addressing CM, two approaches have emerged (Landers et al., 2018). Behavioral skill-based interventions utilize principles from cognitive behavioral therapy (CBT) and social learning theory (Landers et al., 2018; Melendez-Torres et al., 2019). These interventions provide caregivers with explicit strategies for managing and modifying challenging behaviors, such as behavioral monitoring, cognitive restructuring, modeling, positive reinforcement, and negative punishment. PCIT, Keeping Foster Parents Trained and Supported (KEEP; Chamberlain et al., 1992; Price et al., 2009), and Multidimensional Treatment Foster Care (MTFC; Chamberlain, 2003) are some examples of behavioral skill-based interventions used in this population.

In contrast, relational interventions emphasize improving caregiver-child relationship dynamics to support children’s optimal development, rather than teaching specific behavioral skills. Notable relational interventions are rooted in attachment theory, which seeks to improve caregiver-child attachment security and enhance children’s psychosocial development by enhancing parental empathy and sensitivity to their child’s cues as well as fostering caregivers’ reflective capacity (Jugovac et al., 2022).

## Attachment Theory as a Framework for Child Maltreatment Intervention

Attachment theory, initially proposed by Bowlby (1969, 1988), suggests that infants need their caregivers to offer consistent and attuned care, attention, recognition of their emotional states, and support in regulating their emotions. In the presence of attachment figures who can interpret and respond to infants’ signals and emotional needs, infants form secure-autonomous attachments and construct a positive internal working model (IWM). The IWM is one’s schema or generalization of their self-worth and expectations about others, especially those in close relationships. Children who develop positive IWMs have a strong belief in their self-worth and capabilities, while also perceiving those close to them as reliable and trustworthy. Consequently, secure attachment and positive IWMs are associated with adaptive psychosocial outcomes across developmental stages (e.g., Delgado et al., 2022; Pietromonaco & Beck, 2019; Widom et al., 2018).

However, maltreated infants and children often exhibit insecure (i.e., avoidant or anxious-resistant/ambivalent attachment; Ainsworth, 1979) or disorganized attachment patterns with negative IWMs (Baer & Martinez, 2006; Main & Solomon, 1990; Pickreign Stronach et al., 2011). Avoidant attachment, often formed in response to emotionally unavailable parenting and neglect, is linked to an IWM characterized by difficulty trusting others, self-reliance, minimization and devaluation of attachment needs, and fear of intimacy due to discomfort or potential rejection. Children and adults with avoidant attachment have stress regulation challenges when they fail to suppress emotions, contributing to adverse health outcomes (Mikulincer & Shaver, 2019).

Conversely, inconsistent and unpredictable caregiving that only sporadically meets children’s emotional needs often leads to anxious-resistant/ambivalent attachment (Ainsworth, 1979). Individuals with anxious-resistant/ambivalent attachment are often preoccupied with their caregiver relationships but struggle to derive comfort from them. They face difficulty trusting relationship consistency, exhibit high sensitivity to rejection, and often experience doubts about their self-worth. This IWM has been linked with hyperactivation of physiological distress systems and worse health outcomes (Mikulincer & Shaver, 2019).

Lastly, many infants and children who have frightening or traumatizing caregiving experiences develop disorganized attachment (Main & Solomon, 1990), marked by difficulty enacting strategies for seeking comfort and coping. Characteristics include mixed feelings toward caregivers and distrust in others that are highly linked with affect dysregulation, psychopathology, and behavior problems (Pickreign Stronach et al., 2011).

Thus, among families exposed to CM, caregiver–child interaction and attachment are often viewed as primary targets of potential intervention efforts. Psychoeducation on attachment patterns and IWMs fosters caregiver reflection on their own childhood experiences and provides insight into their role in providing consistent and sensitive parenting. Moreover, attachment-based interventions provide training for parents to recognize, interpret, and respond promptly to their children’s needs, often with the help of technology such as video feedback (e.g., Dozier et al., 2006; van der Asdonk et al., 2020). Therefore, attachment-based interventions are posited to effectively mitigate CM’s negative impact on children’s well-being by improving parenting and caregiver-child relationships.

## Past Research on the Effectiveness of Attachment-Based Interventions

Previous systematic reviews and meta-analyses have examined the effectiveness of the attachment-based interventions in improving parenting behaviors and caregiver-child attachment security (Kohlhoff et al., 2022; Mountain et al., 2017). These studies have found that attachment-based interventions were generally effective at improving caregiver sensitivity and attachment security with higher rates of secure attachment and lower rates of disorganized attachment than passive (e.g., care-as-usual) and active control groups.

Beyond caregiver–child relational outcomes, attachment-based interventions have also demonstrated benefits for children’s psychosocial functioning, although important gaps remain in the literature with varying findings across studies and outcome domains. A meta-analysis by Jugovac et al. (2022), which included both attachment-based and emotion-focused interventions, found that these approaches outperformed waitlist controls in reducing child behavioral problems. In two head-to-head trials, attachment-based interventions demonstrated effects comparable to behavioral parenting interventions for reducing externalizing symptoms, indicating similar efficacy across approaches.

Reviews of specific attachment-based interventions further highlight heterogeneity in outcomes. For example, Video-Feedback Intervention to Promote Positive Parenting and Parent-Infant Psychotherapy showed no differences in reducing children’s behavioral problems compared to control conditions (Bakermans-Kranenburg et al., 2023; Barlow et al., 2015). On the other hand, ABC demonstrated promising results for improving emotion regulation and addressing behavioral problems when compared to the control group (Grube & Liming, 2018), with one study examining affect expression during caregiver–child interaction (Lind et al., 2014) and another study using a caregiver-reported questionnaire for behavioral problems (Sprang, 2009).

Notably, despite the growing evidence for the effectiveness of attachment-based interventions, only one systematic review to date has specifically investigated the effectiveness of attachment-based interventions among foster and adopted families with potential histories of CM (Kerr & Cossar, 2014). Despite some evidence for improvements in behavioral outcomes, the study did not include youth’s cognitive functioning and physical health outcomes. Additionally, an updated systematic review is needed to synthesize child well-being outcomes from attachment-based interventions in the past decade.

## Current Study

This systematic review examined the effectiveness of attachment-based parenting interventions in improving maltreated children’s emotional, behavioral, physiological, cognitive, and physical health outcomes. We investigated whether these interventions outperform alternatives, such as usual care or other behavioral skill-based interventions, and identified key characteristics of attachment-based interventions that contribute to positive psychosocial child outcomes.

## Methods

The current systematic review followed the Preferred Reporting Items for Systematic reviews and Meta-Analyses (PRISMA) guidelines (Page et al., 2021). Electronic database searches were conducted using APA PsycInfo, PubMed, CINAHL, ERIC, and Web of Science databases up to April 2025. The following search terms, suggested by previous reviews and meta-analyses (e.g., Kerr & Cossar, 2014; van der Put et al., 2018), were used: (attachment OR attachment theory) AND (child abus* OR child maltreat* OR child neglect*) AND (preven* OR interven*) AND (quasi experiment OR randomized control* OR trial OR RCT interven* OR evaluat* OR experiment* OR effectiveness OR efficacy). Additionally, a snowball technique (Wohlin, 2014) was utilized, involving screening reference lists of included studies both backward (i.e., studying reference sections to identify additional studies) and forward (i.e., evaluating studies citing the selected studies).

## Inclusion and Exclusion Criteria

### Population

Studies were included if caregivers and children were involved in child welfare programs or identified by child protective services (CPS) for experiencing or being at-risk for CM. All primary caregivers, including biological, adopted, foster, and kinship parents, were included.

### Design

Included studies employed quantitative evaluative designs, encompassing quasi-experimental design comparing matched samples and open trial with pre-intervention, post-intervention, and longitudinal follow-ups, as well as randomized controlled trials (RCTs). We excluded single-case designs and intervention studies that did not conduct quantitative analyses.

### Intervention

Interventions that explicitly described Bowlby’s (1969, 1988) and/or Ainsworth’s (1979) theories and research as guiding principles were included. These interventions aimed to (a) increase the caregiver’s understanding of attachment theory, (b) improve the attachment relationship between caregivers and their children, and/or (c) enhance the caregiver’s ability to manage their child’s difficulties using attachment theory. These interventions often emphasized the importance of the caregiver–child relationship as foundational to the development of the child’s internal working model and worldview. They supported caregivers in practicing their role as a secure base and safe haven by allowing children to explore safely while maintaining a sense of security. Lastly, the interventions taught caregivers to attend to children’s distress cues and co-regulate by scaffolding children’s emotional experiences with nonthreatening care. These criteria and definitions were highlighted and further explained in previous attachment-based intervention studies (Kerr & Cossar, 2014) and the Handbook of Attachment-based Interventions (Steele & Steele, 2019).

### Intervention Outcomes

Included studies measured children’s emotional, behavioral, physiological, cognitive, and physical health outcomes. Studies that solely focused on observational or narrative measures of the parental behavior and caregiver–child relationship, such as revictimization of CM, caregiver-child attachment, caregiver sensitivity, and child interactive behavior with caregivers (e.g., child’s engagement with caregiver) were excluded, as these parental and relational outcomes were often the focus of the previous systematic review and meta-analytic studies (e.g., Kohlhoff et al., 2022; Mountain et al., 2017). However, studies utilizing standardized observational measures of infants and toddlers’ mental and/or psychomotor development were included, as they extend beyond the evaluation of relational outcomes.

## Selection of Studies

The articles were screened by the first author. After the initial screening of the titles and abstracts, the first author retrieved full texts of articles to fully determine whether the articles met the inclusion criteria. We used EndNote as the bibliographic software.

## Quality Assessment

Study quality was assessed using the Joanna Briggs Institute (JBI) revised checklists (Barker et al., 2023, 2024). All RCT studies (including those that incorporated quasi-experimental sub-studies into existing RCTs) were evaluated using a 13-item revised JBI Checklist for Randomized Controlled Trials (Barker et al., 2023), and one quasi-experimental study (Moretti et al., 2020) was evaluated using a 9-item revised JBI Checklist for Quasi-Experimental Studies. The first and second authors independently assessed the quality of 8 studies (27% of the included studies) for interrater reliability, with an ICC of .88. The remaining studies were then coded by either the first or the second author. The percentage scores for acceptability of study quality and violations are reported in the last column of Table 1.

**Table 1:** Summary of studies examining child well-being outcomes in attachment-based interventions among maltreated children

Study	Sample characteristics	Sample size	Child age	Child gender (girls %)	Child race	Study design	Attachment intervention	Control	Intervention outcome	Key findings	Quality of studies (Acceptability %, Violation items)
1. Dozier et al. (2006)	Foster and non-foster parents primarily female caregivers from two U.S. mid-Atlantic cities	164 (ABC n = 30, DEF n = 30, Low-risk n = 104)	25.99 mo. (SD = 5.66, range = 3–39.4) at follow-up	51.22%	Black 63%, White 32%, Biracial 5%	RCT	10-session of ABC	1. 10-session of Adapted DEF 2. Low-risk control (not in foster care)	1 mo. follow-up of diurnal cortisol patterns, behav. problems	ABC group lower cortisol than DEF group, but ns. with low-risk control ABC and DEF groups ns. diff. in behav. Problems Only for ABC, older toddlers had fewer behavioral problems than infants	76.92% Item 9 (reliable outcomes), Item 10 (Incomplete follow up data), Item 11 (ITT)
Dozier et al. (2008)	Same sample cohort as Dozier et al. (2006)	141 (ABC n = 46, DEF n = 47, Low-risk n = 48)	19.66 mo. (SD = 5.11, range = 15–24) at follow-up	48.56%	Black 53.7%, White 31.7%, Hispanic 3.7%, Asians 1.2%	RCT	10-session of ABC	1. 10-session of Adapted DEF 2. Low-risk control (not in foster care)	Post-intervention of cortisol patterns during caregiver–child separation	ABC and low-risk control groups lower cortisol than DEF group	76.92% Item 3 (baseline similarities), Item 10 (Incomplete follow up data), Item 11 (ITT)
Lewis-Morrarty et al. (2012)	Same sample cohort as Dozier et al. (2006)	61 (ABC n = 17, DEF n = 20, Low-risk n = 24)	60.3 mo. (SD = 8.6, range = 48–72) at follow-up	50.20%	Black 42.6%, White 36.1% Hispanic, Asians, or Biracial 21.3%	RCT	10-session of ABC	1. 10-session of Adapted DEF 2. Low-risk control (not in foster care)	3 years. follow-up of cog. flexibility, theory of mind (ToM)	ABC and low-risk control groups better cog. flexibility than DEF group ABC group better on ToM than DEF group. Low-risk group marginally better on ToM than DEF group	69.23% Item 3 (baseline similarities), Item 10 (Incomplete follow up data), Item 11 (ITT), Item 12 (appropriate statistics – low power)
Bernard et al. (2017)	Same sample cohort as Dozier et al. (2006)	52 (ABC n = 24, DEF n = 28)	39.52 mo. (SD = 2.98, range = 34.2–46.4) at follow-up	55.77%	Black 55.8%, White 28.8%, Biracial 7.7%, Hispanic 5.8%, Asian 1.9%	RCT	10-session of ABC	10-session of Adapted DEF	2 years. follow-up of receptive language	ABC group higher receptive language than DEF group ABC group better receptive vocab after accounting for gender, placement changes, caregiver edu., income	84.62% Item 10 (Incomplete follow up data), Item 12 (appropriate statistics – low power)
2. Sprang, 2009	Foster parents primarily females recruited from university-based assessment clinic	53 (ABC n = 26, Waitlist n = 27)	42.5 mo. (SD = 18.6, range = 0–5 years.)	84.91%	White 88.7%, Non-white 11.3%	Hybrid RCT^a^	10-session of ABC	Waitlist (with biweekly support clinic services)	Post-intervention of behav. problems	ABC group lower int. and ext. problems than the waitlist controls	76.92% Item 3 (baseline similarities), Item 5 (interveners blind to conditions), Item 12 (appropriate statistics – low power)
3. Bernard et al. (2015a)	Bio. caregivers with allegation of neglect receiving services to divert from foster care in a mid-Atlantic city	101 (ABC n = 49, DEF n = 52)	17.6 mo. (SD = 7.8, range = 5–34.2) at follow-up	42.60%	Black 62%, Biracial 17%, Hispanic 13%, White 8%	RCT	10-session of ABC	10-session of Adapted DEF	2.67 mo. follow-up of diurnal cortisol patterns	ABC group more typical diurnal patterns of cortisol with higher mean log-transformed morning levels and steeper decline across the day than DEF group	92.31% Item 11 (ITT)
Bernard et al. (2015b)	Same sample cohort as Bernard et al. (2015a)	115 (ABC n = 54, DEF n = 61)	50.73 mo. (SD = 4.98, range = 46.5–69.6) follow-up	43.50%	Black 65.2%, Biracial 15.7%, Hispanic 10.4% White 8.7%	RCT	10-session of ABC	10-session of Adapted DEF	3 yrs. follow-up of diurnal cortisol patterns	Same finding as Bernard et al. (2015a) but longer outcome timepoint	92.31% Item 11 (ITT)
Bick et al. (2019)	Same sample cohort as Bernard et al. (2015a)	181 (ABC n = 47, DEF n = 58, Low-risk n = 76)	8.45 years. (SD = 0.35, range = 9–13) at follow-up	48.35%	Black 59.1%, Biracial 21.5%, Hispanic 21.5% White 20.4%	RCT	10-session of ABC	1. Adapted DEF 2. Low-risk control from community centers/schools (matched gender, race)	6 years. follow-up of EEG profiles indicative of cortical delays/immaturity	ABC and low-risk controls had greater high-frequency power (beta, 12–20 Hz) than DEF group, indicating that ABC intervention supports more normative patterns of neural function	92.31% Item 11 (ITT)
Tabach-nick et al. (2019)	Same sample cohort as Bernard et al. (2015a)	96 (ABC n = 43, DEF n = 53)	9.45 years. (SD = .34) at follow-up	48%	Black 66.9%, White 21.4%, Hispanic 18.6% Biracial 11.7%	RCT	10-session of ABC	10-session of Adapted DEF	8 years. follow-up of HR ECG, RSA, skin conductance during caregiver-child recounting stressful event	ABC group lower heart rates and higher RSA than DEF group Ns. difference in skin conductance	76.92% Item 5 (interveners blind to conditions), Item 7 (outcome assessors blind to conditions), Item 11 (ITT)
Garnett et al. (2020)	Same sample cohort as Bernard et al. (2015a)	103 (ABC n = 45; DEF n = 58)	8.52 years. (SD = .67, range = 8–11) at follow-up	45.30%	Black 63.1%, Biracial 15.5% Hispanic 12.6% White 8.7%,	RCT	10-session ABC	10-session of Adapted DEF	8 years. follow-up of diurnal cortisol slope	Ns. effect of ABC intervention on diurnal cortisol slope Post-intervention behavioral coded parental sensitivity mediated the link btw. ABC intervention and diurnal cortisol slope	92.31% Item 11 (ITT)
Valadez et al. (2020)	Same sample cohort as Bernard et al. (2015a)	68 (ABC n = 22, DEF n = 24, Low-risk n = 22)	9.96 years (SD = .84, range = 8.1–12.1) at follow-up	51%	Black 61.8%, Biracial 16.2%, White 11.8%, Hispanic 8.8%	RCT	10-session ABC	1. 10-session of Adapted DEF 2. Low-risk control from community centers/schools (matched gender, race)	8 years. follow-up of fMRI whole-brain analysis of BOLD and amygdala activation to face viewing, behav. problems	ABC group greater BOLD activation to mother (vs stranger) images than DEF group Ns. group diff. in amygdala activation & behav. problems ABC intervention linked with lower behavioral problem via high BOLD reactivity	69.23% Item 5 (interveners blind to conditions), Item 7 (outcome assessors blind to conditions), Item 10 (Incomplete follow up data), Item 11 (ITT)
Valadez et al. (2024)	Same sample cohort as Bernard et al. (2015a)	60 (ABC n = 21, DEF n = 20, Low-risk n = 19)	9.45 years. (SD = .87) at follow-up	50%	Black 61.7% Biracial 16.7% White 10% Hispanic 10%	RCT	10-session of ABC	1. 10-session of Adapted DEF 2. Low-risk control from community centers/schools (matched gender, race)	8 years. follow-up of fMRI PFC BOLD activation, amygdala-PFC BOLD functional connectivity to face viewing (fearful and neutral faces)	Across face viewing, ABC group greater BOLD activation than DEF group. Ns. dif. with low-risk control DEF group showed pos. connectivity btw. amygdala-PFC, while ABC group showed neg. connectivity Sig. indirect effect btw. intervention to BOLD reactivity to face viewing via amygdala-PFC connectivity	69.23% Item 5 (interveners blind to conditions), Item 7 (outcome assessors blind to conditions), Item 10 (Incomplete follow up data), Item 11 (ITT)
Korom et al. (2021)	Same sample cohort as Bernard et al. (2015a)	129 (ABC n = 32, DEF n = 44, Low-risk n = 44)	8. years (SD = .35, range = 8–9.08); 10.57 yrs (SD = .40, range = 9.75–12)	46.51%	Black 60.5%, White 17.1%, Biracial/other 14.7% Hispanic 20.9%	RCT	10-session of ABC	1. 10-session of Adapted DEF 2. Low-risk control from community centers/schools (matched gender, race)	6, 8 years. follow-up of inhibitory control	At age 8, ABC group and low-risk controls sig. better on inhibitory control task than DEF group. Ns. at age 10 At age 10, ns. group diff. on inhibitory control	69.23% Item 2 (concealed allocation), Item 4 (pt. blind to assignments), Item 7 (outcome assessors blind to conditions), Item 11 (ITT)
4. Lind et al. (2017)	Primarily female foster and non-foster care parents with toddlers	173 (ABC n = 63, DEF n = 58, Low-risk n = 52)	47.47 mo (SD = 8.09, range = 30.6–74.2) at follow-up	48.55%	Black 45.7%, White 33.5%, Biracial 9.2%, Hispanic 8.1%, Asian 2.3%	RCT	10-session of ABC-Toddler	1. 10-session of Adapted DEF 2. Low-risk control from university child care center / preschools	1 mo. follow-up of attention problems, cog. flexibility	ABC-T and low-risk control group lower attention problems and better cog. flexibility than DEF group	69.23% Item 2 (concealed allocation), Item 4 (pt. blind to assignments), Item 5 (interveners blind to conditions), Item 11 (ITT)
5. Hoye et al. (2020)	Mothers involved with CPS in a Mid-Atlantic city, U.S	23 (ABC n = 12, DEF n = 11)	14.08 mo. (SD = 4.12, range = 6–21) at baseline	47.83%	Black 52.2%, White 39.1%, Biracial 4.3%, Other 4.3%	RCT	10-session of ABC	10-session of Adapted DEF	1 mo. follow-up of DNA methylation	Methylation values for ABC and DEF groups varied and consistently changed respectively. Functional pathway analyses indicated differences in gene pathways involved in cell signaling, metabolism, and neuronal development	61.54% Item 2 (concealed allocation), Item 4 (pt. blind to assignments), Item 5 (interveners blind to conditions), Item 11 (ITT), Item 12 (appropriate statistics – low power)
6. Spieker et al. (2012a)	Foster (42.4%), bio. (26.7%) or kin (31%) caregivers with a court-ordered placement that resulted in a change in primary caregiver within the prior 7 weeks of pre-intervention	210 (PFR n = 105, EES n = 105)	18.01 mo. (SD = 4.73, range = 10–24) at baseline	56.19%	White 55.2% Black 14.8%, Native American 6.7%, Mixed 19.5%, Pacific Islanders 1%	RCT	10-session of PFR	3-session of EES	Post-intervention, 6 mo. follow-up of caregiver-reported competence, behavioral problems, sleep problems, standardized observation of toddler/infant development	PFR infants higher caregiver-reported competence rating than EES infants in post-intervention Ns. effects on other child outcomes	84.62% Item 5 (interveners blind to conditions), Item 11 (ITT)
Nelson & Spieker, (2013)	Same sample cohort as Spieker et al. (2012a) with foster (37.5%), bio. (37.5%) or kin (25%) caregivers	48 (PFR n = 21, EES n = 25)	17.10 mo. (SD = 4.31, range = 10–25) at baseline	No Report	White 68.8%, Black 16.7%, Native American 12.5%, Pacific Islanders 2.1%	RCT	10-session of PFR	3-session of EES	Post-intervention of morning cortisol level, cortisol during caregiver–child separation task	Ns. treatment effects on morning cortisol level PRF intervention predicted increasing cortisol pattern during post-intervention	84.62% Item 5 (interveners blind to conditions), Item 12 (appropriate statistics – low power)
Oxford et al. (2013)	Same sample cohort as Spieker et al. (2012a) but only bio. parents	43 (PFR n = 18, EES n = 25)	18.21 mo. (SD = 5.01, range = 11–36) at baseline	46.51%	White 67.4%, Mixed 16.3%, Black 9.3%, Hispanic 10%, Native American 7%	RCT	10-session of PFR	3-session of EES	6 mo. follow-up of sleep problems	Ns. group diff. in sleep problems Sig. indirect effect of PFR infants linked with lower sleep problems via lower separation distress	84.62% Item 11 (ITT), Item 12 (appropriate statistics – low power)
Pasalich et al. (2016)	Same sample cohort as Spieker et al. (2012a)	210 (PFR n = 105, EES n = 105)	18.01 mo. (SD = 4.73, range = 10–24) at baseline	56.19%	White 55.2%, Mixed 19.5%, Black 14.8%, Hispanic 10%, Native Amer., 6.7%, Pacific Islander 1%	RCT	10-session of PFR	3-session of EES	Post-intervention, 6 mo. follow-up of infant/toddler problematic behaviors	Mediated moderation effects; Among EES group, placement changes linked with worse attachment security, which in turn was related to worse externalizing problems but not among PFR group	84.62% Item 5 (interveners blind to conditions), Item 11 (ITT)
Oxford et al. (2016a)	Same sample cohort as Spieker et al. (2012a) but only bio. parents	43 (PFR n = 18, EES n = 25)	18.21 mo. (SD = 5.01, range = 11–36) at baseline	46.51%	White 67.4%, Mixed 16.3%, Black 9.3%, Hispanic 10%, Native American 7%	RCT	10-session of PFR	3-session of EES	Post-intervention, 6 mo. follow-up of caregiver-report of competence, behav. problems, standardized observation of emotion regulation	PFR infants higher caregiver-reported competence than EES infants in post-intervention. Ns. diff. in other child outcomes	76.92% Item 5 (interveners blind to conditions), Item 11 (ITT), Item 12 (appropriate statistics – low power)
7. Oxford et al. (2016b)	Bio. parents primarily females from 3 counties with CPS involvement for CM allegation	247 (PRF n = 124, Referral n = 123)	16.37 mo. (SD = 4.46, range = 10–24) at baseline	46.15%	White 61.9%, Hispanic 31.6%, Mixed 31.2%, Black 4%, Asian 2%, Pacific Islander 1%	RCT	10-session of PFR	Referral	Post-intervention, 3 and 6 mo. follow-up of caregiver-report of competence, problem behaviors, standardized observation of toddler/infant dev	PRF group showed lower atypical affective communication than referral group. Ns. diff. in other child outcomes	84.62% Item 5 (interveners blind to conditions), Item 11 (ITT)
Hash et al. (2019)	Same sample cohort as Oxford et al. (2016b)	247 (PRF n = 124, Referral n = 123)	16.37 mo. (SD = 4.46, range = 10–24) at baseline	46.15%	White 61.9%, Hispanic 31.6%, Mixed 31.2%, Black 4%, Asian 2%, Pacific Islander 1%	RCT	10-session of PFR	Referral	Post-intervention, 3 and 6 mo. follow-up of sleep problems	Ns. direct nor indirect effect of intervention predicting children’s sleep problem via parental sensitivity Only for referral group, predicted sleep problem increased with increased children’s adversities	84.62% Item 5 (interveners blind to conditions), Item 11 (ITT)
Hastings et al. (2019)	Same sample cohort as Oxford et al. (2016b)	59 (PRF n = 29, Referral n = 30)	27.78 mo. (SD = 5.13) at 6 mo. follow-up	45.76%	White 68%, Hispanic 34% Mixed 27%	RCT	10-session of PFR	Referral	Post-intervention, 3 and 6 mo. follow-up of baseline RSA, RSA regulation during stressful task with parents and examiners	Ns. diff. in baseline RSA PFR group smaller decreases in RSA from baseline to stressful task than referral group at 6-mo. follow-up Among PFR infants, greater caregiver sensitivity predicted smaller RSA decrease, whereas among referral group, the opposite	84.62% Item 5 (interveners blind to conditions), Item 11 (ITT)
8. Moretti et al. (2020)	Foster & kin caregivers from teen community health centers in Canada	34	12.72 years (SD = 3.28, range = 8–19)	56%	White 41.2%, Indigenous 32.4%, Mixed/others 17.6%	Pre-post test	9-session of Connect for Foster Parents	NA	Post-intervention of behav. Problems	Ext. problems sig. decrease from pre to post whereas int. problems ns. decrease	55.56% Item 2 (control group), Item 3 (baseline similarities), Item 4 (comparisons similar care), Item 9 (appropriate statistics analysis)
9. Pasalich et al. (2021)	Kinship caregivers (54% grandparents, 23% aunts) of adolescents for at least a month in Canberra, Australia	26 (Connect-KP n = 13, CAU n = 13)	10.58 years (SD = 2.55) at baseline	38.46%	Australian European 58%, Aboriginal or Torres Strait Islander 38%	Pilot RCT^b^	9-session of Connect-KP	CAU in clinic (waitlist)	Post-intervention, 6 mo. follow-up of affect regulation, behav. and emo. adjustment	Connect-KP group lower affect suppression than CAU controls Ns. group diff. in affect dyscontrol, behav. and emo. difficulties, prosocial behav Only the Connect-KP group showed behav. and emo. difficulties below the clinical cut-off at post- intervention and 6-mo. follow-up	92.31% Item 5 (interveners blind to conditions), (Despite low N, mentions pilot RCT)
10. Cicchetti et al. (2011)	Bio. mothers with CPS records for individual or family level CM	143 (CPP n = 32, PPI n = 24, CAU n = 35, Low-risk n = 52)	13.34 mo. (SD = .80) at baseline	50.35%	Minority race 78.8%	RCT	11 mo. of CPP	1. PPI 2. CAU (e.g., CPS monitoring) 3. Low-risk control (receives TANF)	6-mo. mid-intervention, post-intervention, 1 year. follow-up of morning cortisol	The CAU group showed decreasing trajectories of morning cortisol, statistically differing from the other groups (i.e., CPP, PPI, and low-risk control)	61.54% Item 2 (concealed allocation), Item 4 (pt. blind to assignments), Item 5 (interveners blind to conditions), Item 10 (Incomplete follow up data), Item 11 (ITT)
Stronach et al. (2013)	Same sample cohort as Cicchetti et al. (2011)	189 (CPP n = 53, PPI n = 49, CAU n = 35, Low-risk n = 52)	13.31 mo. (SD = .81) at baseline	53%	No child race reported but among mothers 74.6% minority race	RCT	11 mo. of CPP	1. PPI 2. CAU (e.g., CPS monitoring) 3. Low-risk control (receives TANF)	1 year. follow-up of behav. problems	Ns. group diff. in behav. problems	69.23% Item 2 (concealed allocation), Item 3 (baseline similarities), Item 4 (pt. blind to assignments), Item 5 (interveners blind to conditions
11. Moss et al. (2011)	Bio parents referred from CPS (n = 54) and community org. for parenting services/monitoring (n = 13) from Quebec, Canada	67 (AVI n = 35, CAU n = 32)	3.35 years (SD = 1.38, range = 12–71 mo.) at baseline	38.80%	No child or parent race reported	RCT	8-week of AVI	CAU (e.g., monthly visit from social workers)	Post-intervention of behav. problems	Ns. group diff. in ext. nor int. problems Among AVI group, behav. problems decreased with age, and among CAU group, behav. problems marginally increased with age	92.31% Item 11 (ITT)
Dubois-Comtois et al. (2017)	Subsample from the same cohort as Moss et al. (2011)	41 (AVI n = 21, CAU n = 20)	17.76 mo. (SD = 8.96, range = 1–30) at baseline	48.80%	No child or parent race reported	RCT	8-week of AVI	CAU (e.g., monthly visit from social workers)	Post-intervention of standardized observation of toddler/infant development	AVI group higher mental and motor development scores than CAU group	92.31% Item 12 (appropriate statistics – low power)
12. van der Asdonk et al. (2020)	Biological parents primarily mothers in four family residential clinics in the Netherlands	56 (VIPP-SD n = 28, CAU n = 28)	3.48 years (SD = 1.74) at baseline	45%	No child or parent race reported	RCT	4.36 sessions of VIPP-SD	CAU in clinic	10 mo. follow-up of behav. problems	Ns, diff. in behav. problems	84.62% Item 5 (interveners blind to conditions), Item 9 (reliable outcomes)

^a^Randomized pre-post study with control group in clinical setting;^b^Effectiveness-implementation Hybrid Designs

RCT, randomized controlled trial; ABC, attachment and biobehavioral catch-up; CPS, child protective services; DEF, developmental education for families; EEG, electroencephalogram; HR ECG, heart rate electrocardiogram; RSA, respiratory sinus arrythmia; PFC BOLD, prefrontal cortex analysis involving clusters of blood oxygen level-dependent; PFR, promoting first relationships; EES, early education support; Connect-KP, connect—kinship parents; CAU, care as usual; CPP, child-parent psychotherapy; PPI, psycho-educational parenting intervention; TANF, temporary assistance for needy family; AVI, attachment video-feedback intervention; VIPP-SD, video-feedback intervention to promote positive parenting and sensitive discipline

## Results

**Fig. 1:**
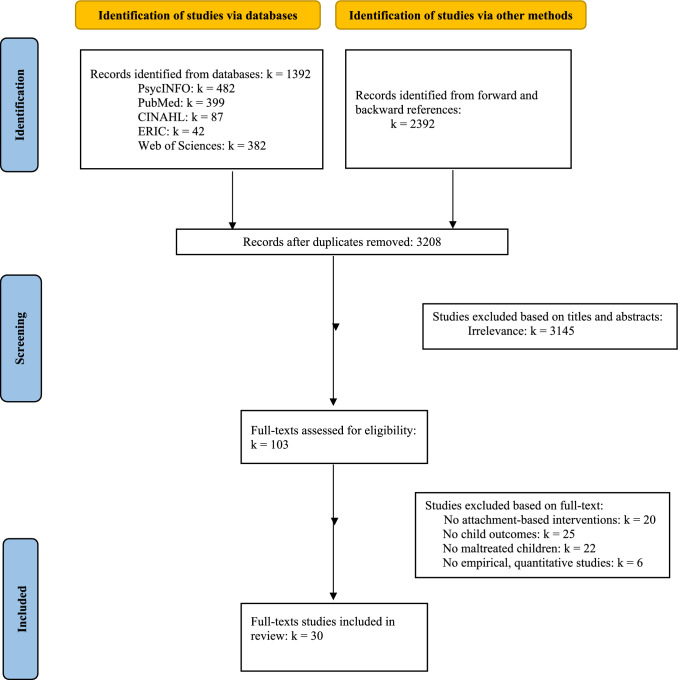
Flow chart of study selection process

A total of *k* = 30 articles met the inclusion criteria. The study selection procedure detailed in Fig. 1 involved 1,051 independent articles from electronic database searches and 2,187 articles from snowballing methods. After screening out 3,145 for irrelevance, 103 full-text articles were assessed, resulting in the final 30 articles.

Out of 30 studies, 24 used six sample cohorts assessing various outcomes, resulting in a total of 12 different sample cohorts. For instance, Dozier et al. (2006) had three follow-up studies using the same sample cohorts (Bernard et al., 2017; Dozier et al., 2008; Lewis-Morrarty et al., 2012), and Bernard et al. (2015a) had seven follow-up studies (e.g., Bernard et al., 2015b; Bick et al., 2019; Korom et al., 2021; Valadez et al., 2020). Similarly, Spieker et al. (2012a) shared the same sample cohort with four other studies (Nelson & Spieker, 2013; Oxford et al., 2013, 2016a, b; Pasalich et al., 2016), and Oxford et al. (2016b) shared the sample cohort with two other studies (Hash et al., 2019; Hastings et al., 2019). Cicchetti et al. (2011) and Stronach et al. (2013) shared the same sample cohort. Lastly, Moss et al. (2011) shared the same sample cohort as Dubois-Comtois et al. (2017). Details of these studies, including sample size, sample characteristics, study design, intervention, control, and key findings, can be found in Table 1. Different sample cohorts are labeled with numbers in Table 1 for clarity.

## Quality Assessment

The quality of studies included in this review is presented in Table 1. The acceptability scores ranged from 55.56% to 92.31%. The study with the lowest acceptability score was the quasi-experimental pre-post study (Moretti et al., 2020), which included a small sample of participants without a control group. Eight RCT studies were in the range of 60%, five studies were in the range of 70%, nine were in the range of 80%, and seven were in the range of 90%. None of the studies achieved 100% acceptability scores, as most studies did not implement or report a triple-blind design, where participants, interventionists, and assessors were blind to the RCT conditions. Additionally, most RCT studies did not conduct intention-to-treat (ITT) analysis. Studies with lower quality should be interpreted with caution.

## Sample Characteristics

The reviewed articles included *N* = 1,423 non-overlapping caregiver-child dyads. Caregiver status, as well as child age, gender, and race are presented in Table 1. Sample sizes for each study were small to moderate, ranging from 23 to 247.

Out of 12 samples, five exclusively consisted of biological caregivers (e.g., Bernard et al., 2015a; Cicchetti et al., 2011; Moss et al., 2011; Oxford et al., 2016b; van der Asdonk et al., 2020), three focused on foster caregivers (e.g., Dozier et al., 2006; Lind et al., 2017; Sprang, 2009), one consisted of only kinship caregivers (Pasalich et al., 2021), two included a mix of various caregivers (Moretti et al., 2020; Spieker et al., 2012a), and one sample did not report the caregiver’s relationship to their child (Hoye et al., 2020). All samples predominantly featured female caregivers, ranging from 85 to 98%. Most samples received attachment interventions during children’s infancy and toddlerhood, except for two study samples, which included children and adolescents (Moretti et al., 2020; Pasalich et al., 2021). At outcome measurement timepoints, the majority of children were in toddlerhood or early childhood except for six studies that used one sample with follow-up assessments extending to middle and late childhood (Bick et al., 2019; Garnett et al., 2020; Korom et al., 2021; Tabachnick et al., 2019; Valadez et al., 2020, 2024). Child gender was generally well-balanced except for two samples that included greater than 60% males (Moss et al., 2011; Pasalich et al., 2021). Six samples were racially/ethnically diverse, with samples comprised of at least 50% non-white children, the highest proportion of whom were primarily Black (e.g., Bernard et al., 2015a; Cicchetti et al., 2011; Dozier et al., 2006; Hoye et al., 2020; Lind et al., 2017; Moretti et al., 2020). Four samples consisted of the majority of White children, ranging from 58 to 89% (Oxford et al., 2016b; Pasalich et al., 2021; Spieker et al., 2012a; Sprang, 2009). Two remaining samples did not report either parent or child race (Moss et al., 2011; van der Asdonk et al., 2020). Most samples were from the U.S. (8 samples), while two were from Canada (Moretti et al., 2020; Moss et al., 2011), one from the Netherlands (van der Asdonk et al., 2020), and one from Australia (Pasalich et al., 2021).

Lastly, out of twelve samples, seven reported the specific maltreatment subtypes that children experienced. Two samples (Bernard et al., 2015a; Dubois-Comtois et al., 2017) reported alleged or substantiated child neglect. Four samples (e.g., Cicchetti et al., 2011; Lind et al., 2017; Moss et al., 2011; van der Asdonk et al., 2020) reported that children experienced various maltreatment subtypes, most commonly with neglect and/or emotional abuse. One study reported that participating children were involved with CPS due to neglect, parental psychopathology, or parental incarceration (Dozier et al., 2006). The rest did not specify the maltreatment subtypes that children experienced.

## Research Design

The majority of studies used traditional or different forms of RCT designs (e.g., pilot RCT, hybrid RCT including implementation outcomes in clinic settings) except for one pre-post comparison test without a control group (Moretti et al., 2020). Additionally, few studies have incorporated quasi-experimental sub-studies into existing RCTs by adding a community sample matched on gender and race (Korom et al., 2021; Lind et al., 2017; Valadez et al., 2020, 2024).

## Attachment-Based Interventions

Six different interventions were examined: (1) Attachment and Biobehavioral Catch-up (ABC; k = 15, five different sample cohorts), (2) Promoting First Relationships (PFR; k = 8, two different sample cohorts), (3) adaptation of Connect (e.g., Connect for Foster Parents, Connect-Kinship Parent; k = 2, two sample cohorts), (4) Child–Parent Psychotherapy (CPP; k = 2, one sample cohort), (5) Attachment Video-Feedback Intervention (AVI; k = 2, one sample cohort), and (6) Video-feedback Intervention to promote Positive Parenting and Sensitive Discipline (VIPP-SD; k = 1).

Most interventions (k = 24), including ABC (with the exception of Sprang, 2009, adapted for use in clinics), PRF, and AVI, were short-term (i.e., 8–10 weeks), home-based parenting interventions for infants and young children. These interventions involved didactics and discussions with caregivers about attachment, sensitive parenting, and infants’ emotion regulation, as well as parenting training with video-feedback, where trainers and caregivers review recordings of caregiver-child interactions and reflect on parenting skills in session. ABC, one of the most widely implemented and empirically supported attachment-based interventions for infants, has also been adapted for early childhood, with promising evidence of effectiveness (California Evidence-Based Clearinghouse for Child Welfare, n.d.-a). It targeted three main components: Caregiver behaviors of nurturance, following the lead with delight, and non-frightening behaviors. It featured professional social workers and psychologists with at least 5 years of clinical experience undergoing a rigorous fidelity process for implementing interventions, including in-the-moment in-vivo coaching, video feedback, and homework. PFR, on the other hand, which is another empirically supported attachment-based intervention, emphasized increasing caregivers’ understanding of children’s needs rather than teaching techniques to extinguish children’s challenging behaviors (California Evidence-Based Clearinghouse for Child Welfare, n.d.-b). The intervention promoted a reflective, collaborative stance, while deliberately avoiding a prescriptive approach. AVI centered around enhancing caregivers’ sensitivity and sense of security through demonstration of caregiver–child interactions, video-feedback, and discussion on attachment and emotion regulation.

Two adapted Connect interventions (Moretti et al., 2020; Pasalich et al., 2021) took distinctive group-based approaches with caregivers of children and adolescents, ages 8–19. The interventions were delivered by trained practitioners in child welfare or community mental health settings. The intervention utilized emotion and attachment-focused discussions, experiential role-plays, and reflective activities to deepen caregiver understanding of trauma, attachment, and adolescent behavioral and mental health challenges. Topics included conflicts arising from relationships with birth parents, feelings of burnout, anxiety, loss, and issues related to placement breakdown and aging out of the system.

CPP used in two studies (Cicchetti et al., 2011; Stronach et al., 2013) was a long-term (i.e., 12 months, although the average range for CPP is typically 20–32 sessions), home-based intervention for infants and young children. CPP adopted a non-didactic, collaborative approach to enhance the physical and perceived safety of families, strengthen family relationships, improve affect regulation for children and caregivers, and increase understanding of trauma. Therapists observed and responded empathically to interactions between mothers and infants, supporting caregivers to challenge distorted perceptions of themselves and their children (e.g., challenging thoughts that “my infant is spoiled” as unresolved resentment from not having their own needs met as a child).

Lastly, VIPP-SD (van der Asdonk et al., 2020), a clinic-based intervention, was brief (i.e., an average of 4.36 sessions) with a behavioral focus. Caregivers of infants learned sensitive disciplinary strategies such as positive reinforcement and sensitive time-outs, which were often emphasized in behavioral skill-based interventions. However, the intervention also explicitly cited attachment theory from Bowlby (1969, 1988) and Ainsworth (1979) with the goal of increasing caregiver sensitivity (Juffer et al., 2018).

## Control Conditions

In 30 studies, four control conditions were employed, often in combinations: (1) active control, (2) comparison groups, (3) care-as-usual or usual care / waitlist, and (4) referrals to resources based on personalized needs.

Active control conditions (k = 21) were most commonly used. Fourteen studies on ABC used a 10-week educational intervention designed to enhance cognitive and linguistic development (Developmental Education for Families, DEF; e.g., Bernard et al., 2015a, b; Dozier et al., 2006). Five PFR studies (Nelson & Spieker, 2013; Oxford et al., 2013, 2016a; Pasalich et al., 2016; Spieker et al., 2012a) featured a 3-session psychoeducational program as a control condition (Early Education Support; EES), connecting families to resources (e.g., Early Head Start, housing, mental health services) and suggesting activities to promote growth and development in infants. Lastly, two studies on CPP used 12-month psychoeducational parenting interventions with CBT techniques as a control condition (Psychoeducational Parenting Intervention; PPI; Cicchetti et al., 2011; Stronach et al., 2013). The clinicians used a didactic approach when addressing concerns related to the socioecological factors of CM, such as limited personal resources, poor social support, and domestic stressors. Other than studies with CPP and PPI, no attachment-based interventions were compared to behavioral skill-based interventions.

Ten studies employed low-risk comparison control groups who were not in foster care (e.g., Bick et al., 2019; Cicchetti et al., 2011; Dozier et al., 2006; Lind et al., 2017). Typically recruited from local community centers and schools, they were often matched for gender and race with the attachment intervention groups (Korom et al., 2021; Lind et al., 2017; Valadez et al., 2020, 2024).

Seven studies had care-as-usual and/or waitlist controls (e.g., Cicchetti et al., 2011; Moss et al., 2011; Pasalich et al., 2021; Sprang, 2009; van der Asdonk et al., 2020). Care-as-usual typically included CPS monitoring or clinical services in clinics. Due to the population’s at-risk nature, no waitlist control was entirely without intervention.

Three studies on PFR (Hash et al., 2019; Hastings et al., 2019; Oxford et al., 2016b) used a referral intervention, with social service providers offering a list of personalized resources (e.g., financial support, educational, housing). This control condition offers less structure and engagement than PFR’s other control condition (i.e., EES).

## Child Psychosocial Outcome Domains and Measures

In assessing attachment-based interventions, two key child outcomes were identified: (1) behavioral problems (k = 11) and (2) physiological and neurological indicators of emotion regulation (k = 12). Behavioral problems, which encompass both internalizing and externalizing problems, were examined across all six attachment-based interventions (i.e., ABC, PFR, CPP, adapted Connect, AVI, and VIPP-SD). Studies utilized caregiver-reported questionnaires, such as the Child Behavioral Checklist (CBCL; Achenbach & Rescorla, 2001) and Strengths and Difficulties Questionnaire (SDQ; Goodman, 1997) at post-intervention and various follow-up time points, ranging from one month to eight years.

Physiological measures of emotion regulation included cortisol level (k = 7), heart rate variability (e.g., respiratory sinus arrhythmia, electrocardiogram; k = 2), and skin conductance (k = 1) measured across days or during stressful caregiver-child interaction or separation tasks. ABC studies predominantly assessed these measures (k = 6; Bernard et al., 2015a, b; Dozier et al., 2006, 2008; Garnett et al., 2020; Tabachnick et al., 2019) at varying follow- up time points ranging from one month to eight years. However, two PFR studies (Hastings et al., 2019; Nelson & Spieker, 2013) and one CPP study (Cicchetti et al., 2011) also utilized cortisol and heart rate variability measures from post-intervention to one year post-intervention. Furthermore, two ABC studies employed functional Magnetic Resonance Imaging (fMRI) to examine prefrontal cortex and amygdala functioning in response to different face stimuli, assessing emotion regulation skills eight years post-intervention (Valadez et al., 2020, 2024).

Four PFR studies (Oxford et al., 2016a, b; Pasalich et al., 2016; Spieker et al., 2012a) and one AVI study (Dubois-Comtois et al., 2017) reported measures related to overall infant and toddler development. The standardized Bayley Scales of Infant and Toddler Development (Bayley, 1993, 2005) assessed children’s psychomotor development, orientation/engagement, and emotion regulation. Additionally, the caregiver-reported Brief Infant Toddler Social and Emotional Assessment (BITSEA; Briggs-Gowan & Carter, 2002) was used to gauge child competence and problem behaviors.

Two ABC studies examined functional (electrophysiological) and molecular (epigenetic) aspects of neurodevelopment (Bick et al., 2019; Hoye et al., 2020). Hoye et al. (2020) examined DNA methylation one month post-intervention to investigate epigenetic factors that can influence gene expression, while Bick et al. (2019) used EEG to understand cortical delays and immaturity eight years post-intervention.

Furthermore, cognitive/executive functioning and linguistic outcomes were measured in four ABC studies (Bernard et al., 2017; Korom et al., 2021; Lewis-Morrarty et al., 2012; Lind et al., 2017). These studies employed different standardized tasks (e.g., Dimensional Change Card Sort; Zelazo, 2006) and a caregiver-reported questionnaire (i.e., CBCL; Achenbach & Rescorla, 2000) to evaluate cognitive flexibility, theory of mind, inhibitory control, and attention problems at varying time points ranging from one month to eight years post-intervention. Receptive language outcome was evaluated two years post-intervention (Bernard et al., 2017).

Lastly, three PFR studies (Hash et al., 2019; Oxford et al., 2013; Spieker et al., 2012a) measured sleeping problems among infants and toddlers through caregiver-reported questionnaires (e.g., CBCL; Achenbach & Rescorla, 2000).

## Findings on Effectiveness of Attachment-Based Interventions

### Attachment and Biobehavioral Catch-up (ABC)

ABC studies found favorable results across different child outcomes compared to the active control conditions (i.e., DEF; k = 14) and waitlist control group (k = 1). Although initial findings indicated significant between-group differences, these differences were no longer significant for several child outcomes (e.g., problem behaviors, diurnal cortisol slope, skin conductance, inhibitory control) at the eight years post-intervention follow-up, which was the last follow-up included in this review.

Four studies produced mixed findings on problem behaviors. Although two studies with distinct sample cohorts of toddlers found lower caregiver-reported internalizing and externalizing problems in children who received ABC compared to those who received DEF (Dozier et al., 2006) and waitlist control group (Sprang, 2009), these differences were not significant among infants (Dozier et al., 2006) and children in middle childhood after eight years of intervention (Valadez et al., 2020).

Regarding physiological and neurological outcomes that measured emotion regulation, eight ABC intervention studies generally showed favorable results compared to DEF. Across different samples and various timepoints, children in the ABC intervention group had lower cortisol, lower heart rates, and higher RSA at rest and during stressful parent–child interactions, as well as more typical diurnal cortisol secretion than those in the DEF intervention group (Bernard et al., 2015a, b; Dozier et al., 2006, 2008; Tabachnick et al., 2019). These outcomes were similar to those of low-risk control groups in the studies. Noteworthy exceptions were the non-significant association between ABC and middle childhood diurnal cortisol slope (Garnett et al., 2020), as well as the non-significant difference between ABC and DEF on skin conductance during stressful parent–child interaction eight years post-intervention (Tabachnick et al., 2019). However, at this time point, two ABC studies that employed fMRI to assess emotion regulation skills found promising neurological outcomes indicative of enhanced sociocognitive development (Valadez et al., 2020, 2024). Children who received ABC displayed greater prefrontal cortex responsivity to maternal cues compared to stranger cues in their middle childhood, which was in turn related to lower caregiver-reported behavioral problems (Valadez et al., 2020). Also, children who received ABC showed higher prefrontal cortex activity and lower amygdala activity when viewing different emotional faces (e.g., fearful, neutral) compared to those who received DEF, which is a pattern more commonly seen in adulthood compared to middle childhood or adolescence (Valadez et al., 2024).

Two other studies with genetic and neurological measures found differences between ABC and DEF. A preliminary DNA methylation study with a small sample (N = 23) suggested consistently altered methylation values, indicating the involvement of different gene pathways related to cell signaling, metabolism, and neuronal development at one month post-intervention. An EEG study during middle childhood suggested that children who received ABC exhibited higher activation in the high-frequency power band at rest compared to those who received DEF, reflecting a neural pattern similar to the low-risk comparison group.

Lastly, four studies with three different samples found overall favorable cognitive and linguistic outcomes associated with ABC compared to DEF, which was adapted to solely focus on cognitive development. During toddlerhood, children in the ABC intervention group had higher receptive language scores than those in the DEF intervention group, after accounting for several key covariates (e.g., caregiver education, income, placement changes, child gender; Bernard et al., 2017). Two studies with children in early childhood (i.e., ages 4–6) found that children who received ABC during infancy and toddlerhood performed better on cognitive flexibility and theory of mind tasks than those who received DEF with lower caregiver-reported attention problems (Lewis-Morrarty et al., 2012; Lind et al., 2017). These children who received ABC showed similar levels of cognitive performance to those in the low-risk comparison control group. During middle childhood, children who received ABC and children from the low-risk comparison group at age eight performed better on inhibitory control tasks than those who received DEF. However, at age 10, there were no significant differences in inhibitory control among ABC, DEF, and the low-risk comparison groups.

### Promoting First Relationships (PFR)

There were mixed results of PFR interventions in improving child outcomes. Out of eight studies that used PFR, seven studies found some nonsignificant main differences in child outcomes between PFR and control conditions (Hash et al., 2019; Hastings et al., 2019; Nelson & Spieker, 2013; Oxford et al., 2013, 2016a, b; Spieker et al., 2012a). These *ns* child outcomes include standardized observational measures of infant and toddler development (Bayley, 1993, 2005), behavioral problems, physiological measures (e.g., morning cortisol), and sleep problems. The control conditions included a lower-dose, 3-session psychoeducational control condition (i.e., EES intervention) and a referral condition during post-, 3-month, and 6-month follow-up assessments. Furthermore, one study found evidence that youth in the PFR group had increased cortisol levels during the parent–child separation compared to the EES group (Nelson & Spieker, 2013). On the other hand, other studies found significant differences in child outcomes between PFR and control conditions, including higher ratings of caregiver-reported infant and toddler competence (e.g., sustained attention, compliance, social relatedness) in the PFR group than the EES group during the post-intervention assessment (Oxford et al., 2016a, b; Spieker et al., 2012a). In addition, alterations in physiological markers indicative of better emotion regulation were observed 6 months post-intervention among the PFR group compared to the EES group, including smaller decreases in RSA from baseline to laboratory challenge tasks (Hastings et al., 2019). Additional significant findings were indirect effects, such as children in the PFR condition exhibiting a reduced likelihood of separation distress, linked to fewer caregiver-reported sleep problems (Oxford et al., 2013). Finally, only among children in control conditions (i.e., EES intervention and referral condition), adversities (e.g., changes in placement) led to worse outcomes (e.g., increased caregiver-reported sleep problems, higher externalizing problems via worse attachment security) but not among those in the PFR condition (Hash et al., 2019; Pasalich et al., 2016).

### Adapted Connect

There were promising preliminary outcomes observed for adapted Connect interventions in two small sample cohorts with foster and kinship care parents (*N*s = 26–34; Moretti et al., 2020; Pasalich et al., 2021). In a within-subjects open pre-post trial with foster parents, caregiver-reported externalizing problems decreased significantly, while internalizing problems showed no significant change (Moretti et al., 2020). In a separate pilot RCT study with kinship care parents, both the Connect-Kinship Parents group and the care-as-usual group showed clinically elevated mean scores of behavioral and emotional difficulties at baseline, but only the Connect-Kinship Parents group demonstrated mean scores below the clinical cut-off at post-intervention and 6-month follow-up (Pasalich et al., 2021). Additionally, at post-intervention and 6-month follow-up, the Connect-Kinship Parents group maintained significantly lower caregiver-reported affect suppression compared to the care-as-usual group. However, most of the youth outcomes (e.g., behavioral and emotional difficulties, prosocial behaviors, affect dyscontrol) were not significantly different between groups when controlling for baseline.

### Child–Parent Psychotherapy (CPP)

No significant child outcomes were reported from two CPP intervention studies with the same sample cohort. At one year post-intervention, there were no group differences in child behavior problems among four groups: (1) CPP, (2) PPI (i.e., an active control receiving psychoeducational parenting intervention using CBT techniques), (3) maltreated care-as-usual, and (4) low-risk comparison control group (Cicchetti et al., 2011). Additionally, no group differences were observed among CPP, PPI, and low-risk comparison control groups in trajectories of cortisol regulation, measured by morning cortisol level at four different timepoints (i.e., pre-, mid-, post-intervention, and one-year follow-up; Stronach et al., 2013).

### Attachment Video-Feedback Intervention (AVI)

Promising preliminary post-intervention outcomes were reported in two AVI intervention RCT studies using one sample cohort with small sample sizes (*N* = 41–67; Dubois-Comtois et al., 2017; Moss et al., 2011). For older toddlers, caregiver-reported behavior problems decreased following AVI, whereas behavior problems marginally increased among the care-as-usual control group (Moss et al., 2011). Furthermore, children who received AVI exhibited higher mental and motor development scores on standardized observational measures of infant and toddler development compared to those in care-as-usual conditions (Bayley, 1993).

### Video-feedback Intervention to Promote Positive Parenting & Sensitive Discipline (VIPP-SD)

Lastly, a study with VIPP-SD intervention with a small sample size (*N* = 56; van der Asdonk et al., 2020) found no difference in caregiver-reported behavior problems between toddlers who were in the VIPP-SD intervention and care-as-usual groups.

## Discussions

This systematic review assessed the effectiveness of attachment-based parenting interventions, an alternative to interventions based on CBT and social learning theories, for improving children’s emotional, behavioral, physiological, cognitive, and physical health outcomes among children who experienced CM. Thirty studies were synthesized, focusing on six distinct attachment-based parenting interventions (ABC, PFR, adapted Connect, CPP, AVI, VIPP-SD). ABC (k = 15) and PRF (k = 8) were prominently represented, with ABC studies including multiple follow-up assessments extending up to eight years post-intervention across five different sample cohorts. The majority of other studies had relatively small sample sizes (i.e., *N* < 60 except for CPP studies) and follow-up assessments up to a year post-intervention. Several interventions (i.e., ABC, PFR, AVI) involved short-term, home-based programs for caregivers of infants and toddlers using psychoeducation, didactics, and video feedback coaching. However, interventions varied in terms of duration (e.g., CPP with a 12-month intervention), approaches (e.g., PFR avoiding “expert” stance to reduce children’s challenging behaviors and CPP with a non-didactic approach), target population (e.g., adapted Connect for foster and kinship care caregivers of adolescents), and setting (e.g., VIPP-SD in a clinic setting, adapted Connect in a group-based setting).

Overall, evidence of effectiveness for enhancing children’s psychosocial outcomes varied. ABC intervention studies showed great promise for enhancing children’s emotion regulation and cognitive outcomes compared to an active control intervention focused on cognitive and linguistic development. Many of the child outcomes in ABC studies were similar to those of non-foster, low-risk children. Although many improvements in children who received ABC were sustained over time, some improvements related to behavioral problems, physiological markers of emotion regulation, and executive functioning were no longer significant after eight years post-intervention. Nevertheless, ABC findings were noteworthy considering the short-term nature of the intervention administered during infancy and toddlerhood.

There was generally limited evidence of effectiveness for the other interventions due to non-significant findings and small sample sizes. Promising initial results were reported for AVI and adapted Connect when compared to the passive, care-as-usual controls. AVI with similar durations, delivery methods, and approaches as ABC showed positive outcomes involving overall infant and toddler development and behavior problems. For kinship and foster caregivers of adolescents, adapted group-based Connect may be beneficial, as discussions and role plays addressing foster parenting for adolescents showed encouraging outcomes related to caregiver-reported behavioral problems and emotion regulation outcomes. However, these studies were conducted with small samples, and replication with larger samples and other active control conditions is necessary to definitively assess the effectiveness of the interventions.

Studies on PFR, CPP, and VIPP-SD interventions did not reveal significant differences in child outcomes related to behavior problems, overall infant and toddler development, and sleep problems when compared with control conditions. For CPP and VIPP-SD, intervention durations (i.e., 12 months for CPP and 4 sessions for VIPP-SD) may have posed challenges in maintaining caregiver engagement, enhancing caregiver-child relationships, and improving child outcomes. For PFR, while caregiver ratings of social‑emotional competence improved, studies found nonsignificant direct effects on behavioral problems, sleep problems, and emotion regulation compared to low-dose controls (e.g., 3-week psychoeducation sessions, referrals). Although the PFR shares a similar duration, target population, setting, and approaches with ABC, a key difference between PFR and ABC might have been that PFR purposefully avoided providing active guidance in reducing children’s difficult behaviors, highlighting a collaborative and reflective approach to focus on children’s underlying needs (California Evidence-Based Clearinghouse for Child Welfare, n.d.-b). On the other hand, ABC provided more active, in-the-moment caregiver coaching to describe parents’ behaviors, link behaviors to intervention targets (e.g., following the child’s lead), and indicate outcomes (Caron et al., 2016). CPP, which took a similar approach to PFR, also yielded non-significant findings. Additionally, the difference could have been proficiency in providers, as community-based providers received a 40- to 80-hour training implemented PFR, compared to professional social workers and psychologists with at least five years of experience, who underwent a rigorous training and fidelity monitoring process before implementing ABC.

## Limitations of the Attachment-Based Child Maltreatment Intervention Literature

Unfortunately, no studies directly compared attachment-based interventions with behavioral skill-based interventions (e.g., PCIT, KEEP, MTFC), except for two studies that compared CPP with psychoeducation of CBT skills (Cicchetti et al., 2011; Stronach et al., 2013). This discrepancy may reflect differences in the developmental focus of the target populations. Attachment-based interventions predominantly involved caregivers of infants and young children, whereas behavioral skill-based interventions were generally designed for parents of school-aged children. Thus, no definitive conclusion can be drawn regarding the effectiveness of attachment-based parenting intervention compared with behavioral skill-based interventions.

Relatedly, there is a noticeable lack of attachment-based interventions designed for maltreated school-aged children in early and middle childhood, apart from adapted Connect programs. Although attachment research has predominantly focused on infancy and toddlerhood, attachment remains a crucial construct across later developmental stages (Opie et al., 2021). In early and middle childhood, children are expected to be more autonomous and demonstrate emotional communication in different settings, such as school. In adolescence, youth are expected to maintain and foster connections with more independence and form their identities. Attachment-based interventions can be mapped to help families achieve key developmental tasks, especially among those with CM histories, who have endured disruptions in attachment relationships. Moreover, female caregivers comprised the majority of samples of the included studies (i.e., 85–98% of samples). Studies should attempt to involve male caregivers, such as fathers.

Lastly, multiple studies included in this review utilized the same sample cohorts. Although replication and follow-up studies provide opportunities to confirm or refute initial findings, they may also inflate the significance of findings and decrease generalizability. Moreover, attachment-based interventions should broaden their focus beyond improving caregiver–child attachment and incorporate an examination of children’s well-being as key outcomes. This is particularly relevant for children with maltreatment experiences who are at risk for myriad socioemotional, cognitive, and health problems (e.g., Lippard & Nemeroff, 2020).

## Direction for Future Research

Researchers should compare the effectiveness of attachment-based interventions with other evidence-based interventions, such as behavioral skill-based interventions, among families with CM histories. Additional research with diverse populations of different ages and larger sample sizes in various settings is also needed to examine the effectiveness of attachment-based parenting interventions for improving child outcomes. For recruitment, future studies should include more male caregivers and consider tailoring interventions for caregivers with similar experiences (e.g., birth caregivers, kinship caregivers, foster care caregivers), given their distinct needs and trauma histories (e.g., Connect for Kinship Care; Pasalich et al., 2021), despite recruitment challenges and concerns with low power. Lastly, researchers should conduct extended follow-ups beyond six months or a year for a more comprehensive evaluation of child outcomes, including cognitive (e.g., executive functioning) and physical health (e.g., growth, health status) that were not assessed in included studies.

## Clinical Implications

Attachment-based interventions may be particularly beneficial for families with a history of CM. These interventions emphasize strengthening the caregiver–child relationship through enhanced parental sensitivity, warmth, and attunement, which can be easily integrated with trauma-informed approaches. Considering that CM is associated with a wide range of adverse behavioral, socioemotional, cognitive, physiological, and physical health outcomes, careful evaluation of the effectiveness of attachment-based interventions in improving child outcomes is warranted. Based on our findings and the limitations of the literature, attachment-based interventions may be particularly well-suited for families with young children, although interventions for adolescents appear promising in group settings (e.g., adapted Connect). In general, short-term (i.e., 8–10 weeks) attachment-based interventions that utilized in-the-moment caregiver coaching with rigorous fidelity and training on attachment theory and application were linked with the best child outcomes. When administering attachment-based interventions to families with CM histories, trainers and providers should also tailor interventions to trauma histories and the family’s culture, as well as prioritize safety and stabilization.

## Limitations of the Present Review

Limitations of the current review include the limited scope of the inclusion criteria. While the review targeted families identified as at risk for CM through child welfare services or CPS referrals, we acknowledge that not all at-risk families are detected through these mechanisms. Additionally, by focusing on maltreated children’s socioemotional, cognitive, and health outcomes, the current review excluded other prominent attachment-based interventions that only reported on parental behaviors and caregiver-child relational outcomes, such as Circle of Security (Hoffman et al., 2006). Moreover, other attachment-based intervention studies that focused on children at high risk for adversities (e.g., clinical samples, caregivers with substance abuse problems) were not included in this review. Future systematic reviews and meta-analyses should examine the effectiveness of attachment-based interventions among children at-risk for broader adversities by comprehensively examining caregiver, child, and systemic outcomes, such as child welfare placement outcomes and school adjustment.

Furthermore, some researchers have argued that behavioral skill-based interventions, such as PCIT, should be seen as attachment-based interventions, given that they enhance caregiver-child relationships and the caregiver’s ability to identify children’s emotional and behavioral needs (Allen et al., 2014). However, these interventions have a primary theoretical orientation in CBT and social learning theories, which was the reason they were excluded from this review. Recent efforts to incorporate attachment theories into behavioral skill-based interventions have been reported in the literature (Belanger et al., 2023). For a systematic review and meta-analysis that summarized 81 studies that examined the effectiveness of behavioral skill-based (e.g., PCIT) and attachment-based interventions (e.g., ABC, PFR, VIPP, AVI) among families with CM histories, please see Bergsund et al. (2023). However, it examined the effectiveness of the interventions using observed behaviors during parent–child interactions rather than child outcomes.

## Conclusion

Rigorous, short-term attachment-based parenting interventions that provide active, in-the-moment, in-vivo caregiver coaching show the potential to enhance maltreated children’s psychosocial outcomes. Subsequent research on attachment-based interventions is needed to evaluate their effectiveness comprehensively.

## Data Availability

No datasets were generated or analysed during the current study.
